# Electrical and Electro-Thermal Characteristics of (Carbon Black-Graphite)/LLDPE Composites with PTC Effect

**DOI:** 10.3390/ma17051224

**Published:** 2024-03-06

**Authors:** Eduard-Marius Lungulescu, Cristina Stancu, Radu Setnescu, Petru V. Notingher, Teodor-Adrian Badea

**Affiliations:** 1 National Institute for Research and Development in Electrical Engineering ICPE-CA, 313 Splaiul Unirii, 030138 Bucharest, Romania; radu.setnescu@icpe-ca.ro; 2 Faculty of Electrical Engineering, University POLITEHNICA of Bucharest, 313 Splaiul Independentei, 060042 Bucharest, Romania; petrunot2000@yahoo.fr; 3 Department of Advanced Technologies, Faculty of Sciences and Arts, Valahia University of Târgoviște, 13 Aleea Sinaia, 130004 Targoviste, Romania; 4 Romanian Research and Development Institute for Gas Turbines COMOTI, 220D Iuliu Maniu Av., 061126 Bucharest, Romania; teodor.badea@comoti.ro

**Keywords:** LLDPE composite, PTC, conductivity, self-regulating temperature, carbon filler

## Abstract

Electrical properties and electro-thermal behavior were studied in composites with carbon black (CB) or hybrid filler (CB and graphite) and a matrix of linear low-density polyethylene (LLDPE). LLDPE, a (co)polymer with low crystallinity but with high structural regularity, was less studied for *Positive Temperature Coefficient* (PTC) applications, but it would be of interest due to its higher flexibility as compared to HDPE. Structural characterization by scanning electron microscopy (SEM) confirmed a segregated structure resulted from preparation by solid state powder mixing followed by hot molding. Direct current (DC) conductivity measurements resulted in a percolation threshold of around 8% (w) for CB/LLDPE composites. Increased filler concentrations resulted in increased alternating current (AC) conductivity, electrical permittivity and loss factor. Resistivity-temperature curves indicate the dependence of the temperature at which the maximum of resistivity is reached (T_max(R)_) on the filler concentration, as well as a differentiation in the T_max(R)_ from the crystalline transition temperatures determined by DSC. These results suggest that crystallinity is not the only determining factor of the PTC mechanism in this case. This behavior is different from similar high-crystallinity composites, and suggests a specific interaction between the conductive filler and the polymeric matrix. A strong dependence of the PTC effect on filler concentration and an optimal concentration range between 14 and 19% were also found. Graphite has a beneficial effect not only on conductivity, but also on PTC behavior. *Temperature* vs. *time* experiments, revealed good temperature self-regulation properties and current and voltage limitation, and irrespective of the applied voltage and composite type, the equilibrium superficial temperature did not exceed 80 °C, while the equilibrium current traversing the sample dropped from 22 mA at 35 V to 5 mA at 150 V, proving the limitation capacities of these materials. The concentration effects revealed in this work could open new perspectives for the compositional control of both the self-limiting and interrupting properties for various low-temperature applications.

## 1. Introduction

It is widely known that by introducing a quantity of a conductive material in the form of powder (such as carbon black, graphite, carbon nanotubes, graphene, metallic powders) into a polymeric matrix, the electrical conductivity of the composite material can be approximately 10 orders of magnitude higher than that of the pure polymer [1,2,3,4]. While retaining the valuable properties of polymers—mechanical strength, flexibility, chemical inertness and easy processability—conductive polymer composites (CPCs) currently have numerous applications, with those related to electrical engineering arguably being the most significant [5]. These applications include electrodes for fuel cells and electrochemical sources [6], high-capacitance capacitors [2], high voltage power cable shields [1], screens for the electromagnetic protection of electronic equipment and human beings [7,8], radar wave absorption [2], electronic packaging, antistatic fabrics [2,6,9], temperature sensors, current and voltage protection [10], heating elements, etc. [1,2,11,12,13,14].

As for the polymeric matrix used to obtain CPCs, a wide range of thermoplastic, thermosetting, or elastomeric polymers, either singularly or in blends, is employed [11,15]. Among all polymers, polyethylene, which holds the largest share in synthetic polymer production, and especially HDPE, is most frequently mentioned as the matrix for CPCs. Various materials, metallic, oxide, or carbon-based, are mentioned as conductive fillers [16]. Among them, carbon materials are of greatest interest because they have a lower density than metals or oxides and comparable electrical conductivity, are more stable to corrosion than metals, and are less susceptible to initiating the oxidation of the polymer matrix. Commonly used carbonaceous conductive fillers include carbon black (CB [10,14]), graphite (Gr), carbon fibers (CF), and carbon nanotubes (CNT, MWCNT) [6]. Carbon black and graphite are widely used to prepare conductive composites because they present high electrical conductivity and chemical stability, are relatively inexpensive and have a good commercial availability. Carbon black has a primary structure composed of spherical nanoparticles of around 30 nm size and imparts easy processability to composites [11,17,18]. However, CB particles are often agglomerated into large aggregates due to strong van der Waals forces, which, combined with the unfavorable influence of aspect ratio [19], result in relatively high percolation threshold values (15–20% *w*/*w*). Nevertheless, considerably lower values of the percolation threshold have been reported, even for CB, by creating segregated structures (with conductive material particles unevenly distributed within the polymer matrix), through the use of hybrid fillers or modified particles [20], as well as by using of high-viscosity polymer matrices [11,21].

In general, fillers with high aspect ratio values, such as carbon fibers or CNTs, enable the achievement of low percolation threshold values [19,20]. However, instances of non-percolation are also known, typically caused by weak dispersion of the filler, as seen with acid-treated MWCNTs/PMMA [19].

A more recent trend involves the use of hybrid fillers, consisting of mixtures of particles with different characteristics, especially with different aspect ratio values. Essentially, combining particles with high aspect ratios and other particles (conductive or not) with low aspect ratios and microscopic dimensions creates so-called excluded volumes in the polymeric matrix, with a favorable effect on increasing electrical conductivity and achieving lower percolation thresholds [2]. Such structures are also considered segregated, meaning that conductive particles are unevenly distributed on a microscopic scale around islands of low aspect ratio particles, and electrical conduction can be described by combining percolation theory and the Voronoi geometric model [2].

The methods for dispersing fillers In the polymeric matrix applicable to thermoplastic matrices include melt mixing [19,21,22], solution mixing [9], dry mixing of components [23], in situ polymerization [11] and others. Subsequently, items with the desired shape are usually formed by hot press molding.

A distinct category of CPCs is composed of CPCs that present an increase in electrical resistivity with increasing temperature, i.e., the PTC effect (positive temperature coefficient) and, in particular, an important jump in resistivity (of several orders of magnitude), which results in the transition of the material from a conductor to an insulator. If the temperature exceeds the value corresponding to the maximum resistivity, a decrease in resistivity can be observed (NTC effect). Beyond the temperature corresponding to the maximum resistivity, a decrease in resistivity (*Negative Temperature Coefficient*—NTC effect) can be observed upon further heating.

A plausible explanation of the PTC behavior of these materials is based on depercolation near a structural transition point of the polymer matrix. This effect is very clear in the case of polymer matrices with high crystallinity, such as HDPE, for which the temperature of reaching the maximum resistivity of the composite is very close to or coincides with the peak temperature of the melting endotherm of the polymer [22]. For other systems, the resistivity jump can be correlated with the glass transition, as for example in the case of epoxy matrices, or with the activation of the motion of some polymer chain segments, as in the case of amorphous matrix systems [22]. The amplitude of the resistivity jump depends on the concentration and distribution of the filler material [11]. Such materials, especially those with no or a low NTC effect, are interesting for applications where self-limiting electrical power is involved, such as self-regulating heating elements, current limiters, overcurrent protection, micro-switches, sensors, etc. [18,23].

Other CPCs often exhibit a monotonous decrease in resistivity with temperature. This effect, called NTC (negative temperature coefficient) does not enable the use of materials in self-limiting applications, but makes them useful for other applications, such as resistive temperature sensors [24].

In the design of CPC and PTC materials, the study of the dependence of the electrical conductivity on the concentration of the conductive charge is of particular importance, as it allows the rational dosage of the components as well as the control of the properties of the resulting composites. Typically, the graphical representation of the dependence of *ρ* as a function of the conductive filler concentration ϕ (expressed as either volume or mass fraction) is presented as a sigmoidal curve on which three important regions are distinguished (see Figure S1 in the Supplementary Materials and [19] for typical conductivity or resistivity vs. *ϕ* curves, respectively), namely: (i) an initial region, in which although the content of conductive charge added to the polymer increases, the resistivity decreases very little, so that the material practically remains an insulator; (ii) upon reaching a certain concentration, called the critical concentration *(ϕ_cr_*), the resistivity drops suddenly, by several orders of magnitude, and the material becomes electrically conductive; and (iii) for *ϕ > ϕ_cr_*, increasing in conductive filler concentration results in a slow increase in conductivity. The specific shape of the *σ* (or *ρ*) vs. *ϕ* curves may suggest the mechanism of electrical conduction in the composites. Thus, it is considered that when electric percolation is reached, a sufficiently large number of conductive paths are formed that ensure the passage of the electric current through the sample. A theory [25] considers that all the particles of a conductive path must be in direct physical contact to ensure the continuity of the path, while another theory [26,27] admits that between any two consecutive particles of the conductive phase, a gap (whose maximum width has been estimated at 2 nm) that electrons can traverse through a tunneling effect could exist. In both theories, the conductor–insulator transition specific to PTC materials is due to the appearance of, respectively, an increase in the gap between the conductive particles that leads to the interruption of direct contact, or an increase in the gap width over the tunelation limit. It was demonstrated that either one of the mechanisms can dominate depending on the composition of the material: for high values of the filler concentration *ϕ* > *ϕ_cr_*, the electrical conductivity satisfies the law of electrical conduction and it can be assumed that the conductive particles are in direct contact, while for values of *ϕ* < *ϕ_cr_*, electron tunneling predominates [28].

In this study, the influence of the composition on the DC and AC electrical conductivity properties, the variation of electrical resistivity with temperature, and the electro-thermal effect of a composite with a hybrid conductive filler (CB + Gr) and LLDPE matrix were investigated. To our knowledge, LLDPE is less studied as a matrix for PTC materials, although it could be of interest due to its low crystallinity, high structural regularity, high flexibility and elevated melting temperature, close to that of HDPE [24,29]. We also demonstrated the strong effects of filler concentration on both the PTC intensity and transition temperature (from PTC to NTC) of these materials. The combination of CB and graphite used to impart the electrical conductivity of these materials would be of practical interest due to the economic effectiveness and wide availability of these materials.

## 2. Materials and Methods

### 2.1. Materials

Linear low-density polyethylene (LLDPE) powder with density 0.935 g/cm^3^ and MFI 5.0 m/10 min at 190 °C/2.16 kg (producer data), type RX 806 Natural from Resinex (Bucharest, Romania), was used, in the as-received state, as a polymer matrix. Carbon black (Fast Extruder Furnace—FEF type) and natural graphite flakes (CR10) were used as conductive fillers (see more details in [30]). Irganox 1010 (pentaerythritol tetrakis(3-(3,5-di-tert-butyl-4-hydroxyphenyl) propionate) was used in concentration of 0.05% (*w*/*w*) for matrix stabilization against oxidation. 

The list of abbreviations used in this manuscript is presented in Table 1.

### 2.2. Preparation of Composites and Samples for Measurements

Composites were obtained through hot pressing of physical mixtures of polymer particles (LLDPE) and carbonaceous particles of carbon black and graphite, following a previously described procedure [31]. The dispersion of conductive particles was initially achieved through physical pre-mixing, followed by high-shear solid-state mixing applied to the pre-mixed system. Composite sample formation was conducted by hot pressing (at 160 °C, 6.5 bar) in a rectangular mold (190 × 120 × 0.7 mm) using a hydraulic press. Cooling was performed under pressure at a slow rate of approximately 1 °C/min until reaching 60 °C, followed by pressure release and natural cooling to ambient temperature.

The code names of the prepared composite samples are shown in Table 2. The composition of these materials is also indicated within the table.

The measurement of electrical properties was conducted on plate-shaped samples (with approximate dimensions of 100 × 100 × 0.7 mm) obtained from the plates resulting from molding. The measurements of electro-thermal properties and the temperature dependence of resistivity were performed on specimens with dimensions of 30 × 25 × 0.7 mm, obtained from plates similar to those used for electrical property measurements. Prior to measurements, the extremities of each sample were covered on both faces by conductive silver paste. The width of these conductive traces was 5 mm. Finally, a copper foil was tightly fixed on the silver conductive traces, forming the electrodes of each measured specimen.

### 2.3. Sample Conditioning

Before the electrical measurements, all samples were thermally preconditioned in an oven for 72 h at a temperature of 50 °C. After preconditioning, the thickness of each sample was measured at 5 points (in the center and in the 4 corners). The average value of the thickness of the samples was 0.713 ± 0.028 mm.

A similar pre-conditioning treatment, i.e., 72 h at 30 ± 1 °C, 50 ± 5% r.h., was applied before other measurements described below (namely FTIR, DSC, *R* vs. *T* and *T* vs. *t*, U).

## 3. Instruments and Methods

### 3.1. Scanning Electron Microscopy

The microstructural and morphological analyses of specimens were performed on secondary electron images (ETD detector—Everhart Thornley Detector, FEI Company, Hillsboro, OR, USA) by scanning electron microscopy using a FEI F50 Inspect instrument. The analysis was performed on the specimen’s cross-section under the following conditions: acceleration voltage 10 kV, acquisition time 50 s, spot size 3 nm and working distance 6.0–6.9 mm.

### 3.2. Differential Scanning Calorimetry

DSC thermograms of the blank LLDPE sample and (CB-Gr)/LLDPE composites were recorded using a Setaram 131 evo instrument (Setaram, Caluire-et-Cuire, France), employing 30 μL aluminum crucibles with pierced lids. The mass of each sample was 4.5 ± 0.1 mg, except for the pure LLDPE, which were 3.6 ± 0.1 mg. The samples were heated at a constant rate (10 °C/min) from 35 °C to 200 °C under a nitrogen flow (50 mL/min), after maintaining 5 min at 200 °C, the sample was cooled back under nitrogen at a rate of 10 °C/min till 35 °C. Two such cycles were applied for each sample, and measurements were conducted in duplicate.

On the heating curve the onset temperature (*T_onset_*), peak maximum temperature (*T_m_*), and offset temperature (*T_offset_*) of the endothermic peak were determined, along with the melting enthalpy (Δ*H_m_*). Similarly, on the cooling curve, the parameters of the solidification endotherm were determined, namely the onset temperature (*T’_onset_*), peak maximum temperature (*T_c_*), offset temperature (*T’_offset_*) and the crystallization enthalpy of (ΔH_c_). Throughout the work, especially when comparing *R* vs. *T* data, the terms *T_m(DSC)_* and *T_c(DSC)_* were used instead of *T_m_* and *T_c_*, respectively, in order to emphasize the DSC origin of these data.

The degree of crystallinity (Cr (%)) was calculated using a simple formula [32]:

*Cr (%)* =100·Δ*H_cor_*/Δ*H_100%_* (1)

where Δ*H_cor_* is the latent heat of fusion Δ*H* corrected with the polymer percentage (*p*) in the composite.

Δ*H_cor_* = Δ*H*·*p*/100 (2)

The Δ*H_100%_* = 279 (J/g) represents the melting enthalpy of 100% crystalline polyethylene [32]. The data processing procedure for DSC was described in more detail in previous works [30,31,33].

### 3.3. FTIR Spectroscopy

The infrared spectra were recorded in ATR for both composites and the LLDPE base polymer. The spectral range was 4000–400 cm^−1^, resolution 2 cm^−1^. For each measurement, 48 scans were performed. For the carbon-containing samples, a baseline correction before the main peaks was necessary.

The calculation on the spectra, including peak wavelength, peak absorbance and spectra comparison were performed by specific applications Spectra Manager (Jasco, Tokyo, Japan) and Essential FTIR (Biorad, Tokyo, Japan).

### 3.4. DC Measurements

To determine the direct current (DC) conductivity, an apparatus described earlier [34] was employed. The setup comprised a Keithley model 6517 B electrometer (Keithley Instruments Inc., Cleveland, OH, USA), a Keithley 8009 measurement cell and a PC, supplemented with a Trade Raypa oven with forced air circulation (and adjustable temperature between 30 and 250 °C) in which the measurement cell was placed for measurements at different temperatures.

The values of the absorption currents (*I_a_*) were measured for 600 s, for two different applied voltage values (*U_0_*), namely 1 V and 100 V. The DC electrical conductivity was calculated with the following equation:

(3) $$\sigma_{DC}=\frac{I_{a}}{U_{0}}\times \frac{g}{A}$$

where: *g* is the thickness of the planar sample and *A* = *πD^2^/4* is the area of the upper cylindrical electrode with diameter *D*.

### 3.5. AC Measurements

The experimental determinations of the components of the complex relative permittivites (ε*_r_*′ and ε*_r_*″), complex conductivities (*σ*′ and *σ*″) and the loss factor (tgδ) were performed with an Alpha-A Novocontrol impedance analyzer from Novocontrol Technologies GmbH & Co. KG, Montabaur, Germany. The real part of the complex conductivity (*σ_ca_*) was determined for all samples at the RMS voltage value *U_RMS_* = *U* = 1 V and frequencies ranging from 1 Hz to 500 MHz.

The study of temperature dependence (*T*) of the electrical resistivity (*ρ*) was conducted by measuring the electrical resistance of the specimens (*R*) at varying temperatures, using the experimental setup already presented [32], on specimens removed from the plate, with a 20 mm space between electrodes. Since, in this case, the electrodes were applied across the whole surface at the ends of the specimen, the experimentally measured value was volume resistance. As *ρ_V_ = RA/g*, where *A* is the specimen surface and *g* its thickness, the curves *ρ_V_* vs. *T* and *R* vs. *T* are identical. Hence, below, the curves *R* vs. *T* will be analyzed.

**Figure 1:**
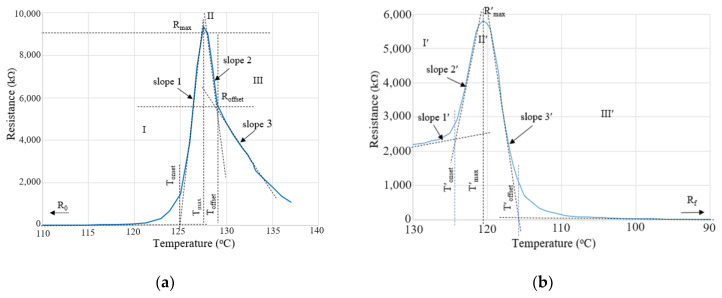
Typical R vs. T heating (**a**) and T cooling (**b**) curve and kinetic parameters.

For obtaining the *R* vs. *T* curves, the specimens were placed in a Memmert oven, and the temperature was programmed to increase at a heating rate of 1 °C/min. The electrical resistance was measured using a multimeter positioned outside the oven and connected to the specimen through conductors. For high resistivity specimens, an insulation tester of type UNI-T UT512 (Uni-trend Technology Co., Ltd., Dongguan, China) was used. Parameters characterizing the variation of resistivity during the heating of the samples are shown in Figure 1.

### 3.6. Measurement of the Electro-Thermal Effect

The measurement of the dependence of the temperature at the surface of the sample on time and applied voltage (either AC or DC), the so called electro-thermal effect, was carried out using a setup similar to the one described earlier [31]. The current temperature (*T*), the equilibrium temperature (*T_eq_*), the intensity of the electric current flowing through the sample (*I*), as well as the intensity upon reaching the temperature equilibrium (I_eq_), were determined.

## 4. Results and Discussion

### 4.1. Structural Characterization

#### 4.1.1. SEM (Scanning Electron Microscopy)

**Figure 2:**
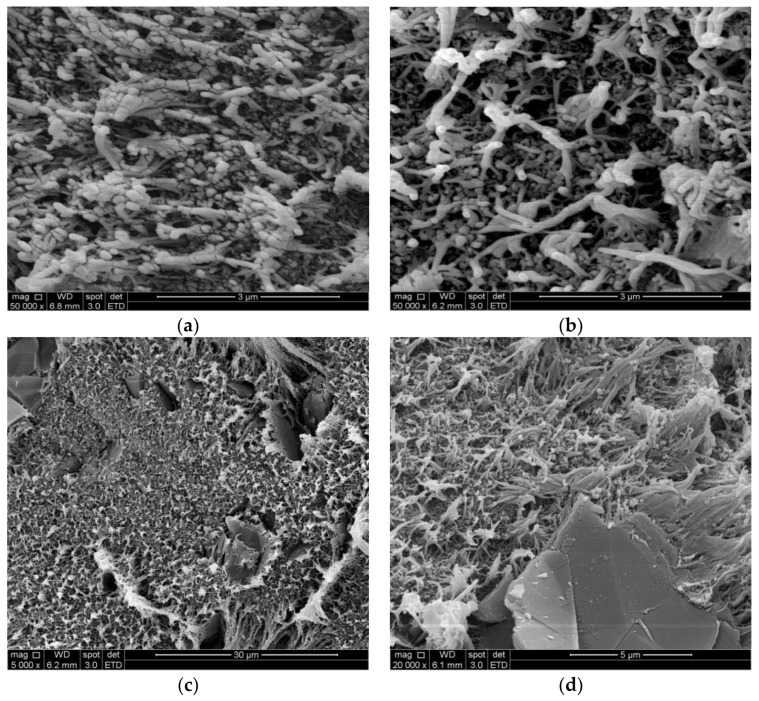
SEM images of some studied composites in a fresh state (unconditioned samples) at different magnifications: (**a**) LLD 190 (50,000×); (**b**) LLD 192 (50,000×); (**c**) LLD 192 (5000×); (**d**) LLD 122 (20,000×).

The morphology of the composites under study is depicted in Figure 2a,b, representing the LLD 190 and LLD 192 composites, respectively. These images can be qualitatively analyzed on the basis of particle shape, dimensions and gray nuance intensities. First of all, the materials show a pronounced micro-heterogeneous character in which the presence of shiny filamentous particles, with dimensions in the order of microns, as well as spheroidal particles with dimensions of 30–100 nm, are observed. The filamentous particles can be assigned to the insulating polymer, while the small spheroidal particles are primary carbon black particles (30 nm) or associations of primary particles (30–100 nm), resulting from the breakdown of the initial aggregates (see a typical image of CB used within this work in reference [30]). If these CB particles are part of a conductive path that allows electrical charges to flow, they appear darker (see for example [35]). Note that during measurement, the sample heats up, so a significant part of the conductive paths could break, resulting in the increased brightness of the CB particles. The graphite particles are not visible in this image, but their presence can be easily observed in lower magnification images (for example, see Figure 2c,d), with dimensions in the order of ten microns, typical for graphite flakes (see a typical SEM image of these particles in reference [30]). Hence, the darker areas in the vicinity of the filiform polymer particles as well as the spheroidal particles are related to conductive or potentially conductive areas belonging to conductive paths within the material.

#### 4.1.2. Differential Scanning Calorimetry

**Figure 3:**
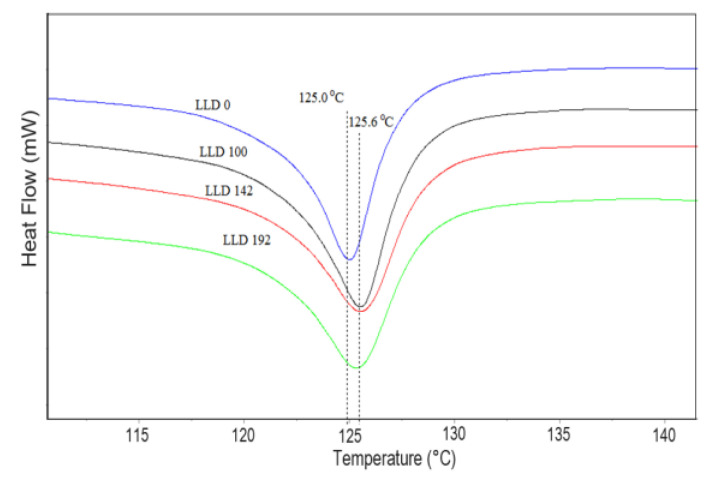
DSC curves of LLDPE and two different (CB-Gr)/LLDPE composites.

**Figure 4:**
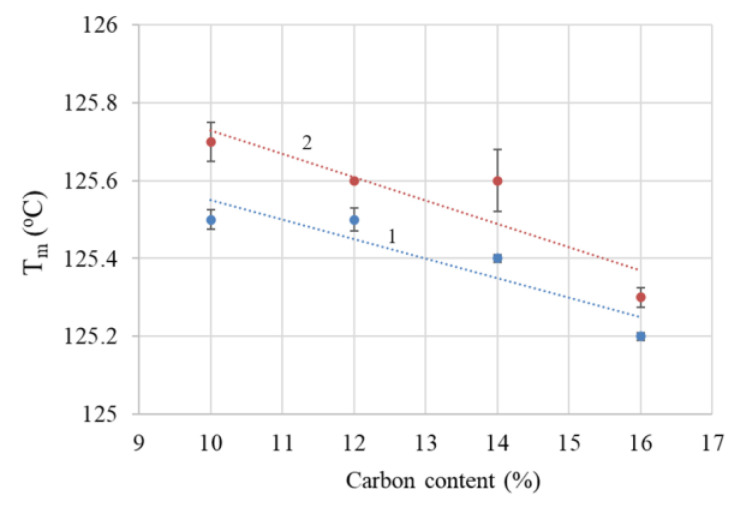
T_m_ decrease with total carbon content: (1) composites with CB; (2) composites with CB and Gr.

The blank polymer sample (LLD 0) presents an endotherm with a peak at 125.8 °C in the heating curves at the first cycle and 125.0 °C at the second cycle, with the difference possibly arising from the sample’s history (mainly due to different solidification conditions during molding and cooling in the DSC instrument, respectively). Similarly, the composites show slightly higher values of T_m_, ΔH_cor_, and crystallinity during the first scan as compared to the second. Besides the previously mentioned cooling conditions, slow crystallization at room temperature within the time span between sample preparation and DSC measurement is also possible. With the second scan, the differences between the parameters of different samples are small, as indicated by the curves shown in Figure 3 and the data in Table S1 from the Supplementary Materials. Thus, the Tm values of the composites have an average of 125.5 °C, with a standard deviation of 0.17. A slight decreasing trend with carbon content can be observed (Figure 4), with lower values for composites with CB as compared to CB + Gr composites. Compared to unmodified LLDPE, all Tm values are slightly higher, suggesting that carbon particles may, to some extent, favor the crystallization of LLDPE, possibly by improving the heat transfer, but increasing carbon content may negatively affect the crystallization process. A similar increase in the crystallinity of LLDPE-based composites was previously reported for different fillers [36,37,38] and was assigned to a possible nucleation effect of the filler [38].

**Figure 5:**
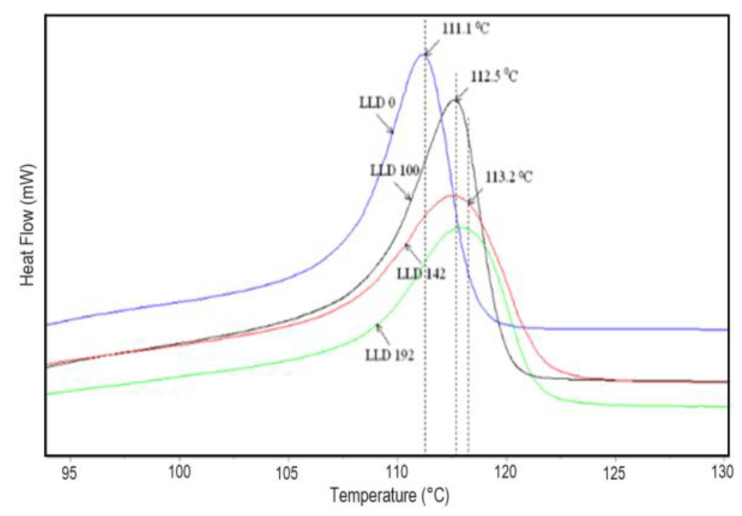
DSC cooling curves of LLDPE (blank) and three different (CB, Gr)/LLDPE composites.

The cooling curves (Figure 5, Table S2) lead to similar conclusions, indicating that the crystallization temperature of the composites is higher than that of the pure polymer. Additionally, the CB and graphite-containing samples present slightly higher crystallization temperatures than those containing CB only. In all cases, the variation of T_c_ with the concentration of conductive filler was very small (the standard deviation of T_c_ values was 0.42 for CB samples, and 0.58 for CB and graphite-containing samples.

The vertical lines indicate the melting temperature of LLDPE (125.0 °C) and the average melting temperature of the studied CB and CB, Gr composites.

#### 4.1.3. FTIR

**Figure 6:**
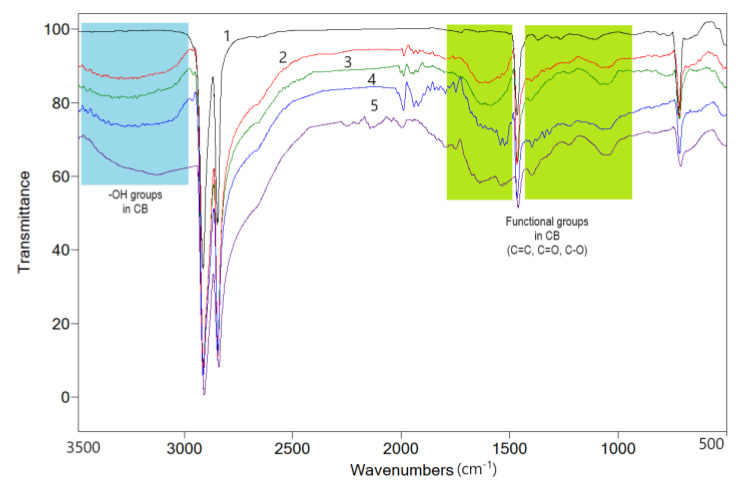
FTIR spectra of polymer matrix and different composites: 1—LLD 0; 2—LLD 120; 3—LLD 190; 4—LLD 122; 5—LLD 192.

The spectrum of the blank LLDPE sample is shown in Figure 6. It is typical for polyethylenes, showing two main bands at 2915 cm^−1^ and 2848 cm^−1^.

As mentioned by Nikishida and Coates [39] regarding the differentiation between low-density polyethylene (LDPE) and linear low-density polyethylene (LLDPE), the bands at 890 cm^−1^ (vinylidene groups) and 910 cm^−1^ (terminal vinyl groups) present very weak intensities in LLDPE, while for LDPE, the band at 890 cm^−1^ is dominant [40]. Indeed, the intensity of these bands in the blank sample is very weak, confirming the LLDPE nature of the polymer. Another band, attributed to CH_3_, specifically occurring at 1378 cm^−1^ (proportional to the number of branches), is also weak in intensity in our case.

The incorporation of CB within the polymer matrix increased optical absorption in the range 3500–3000 cm^−1^, in the form of a broad band with a maximum at ca. 3300 cm^−1^. This wide band shows an increasing trend with the CB content of the samples and is attributed to the -OH groups on the CB surface (oxidized -COOH and -OH groups as well as adsorbed water molecules [41,42,43,44]). The weak band at 2962 cm^−1^ is attributed to C-H groups of the raw material residues from CB synthesis [41], while the bands at 1796, 1740 cm^−1^ (also weak and absent from the matrix spectrum), to oxidized groups on the CB surface. The broad band between 1700 and 1470 cm^−1^ corresponds to different absorptions of the structural elements of CB, as well as to the oxygenated groups occurring on its surface, such as C=C (1640 cm^−1^–graphitization, 1600 cm^−1^), C=O (1680 cm^−1^), C=O chelated with phenolic hydroxyls (1600 cm^−1^), etc. [41,45]). The spectral range between 1420 and 760 cm^−1^ also contains bands that can be assigned to oxygenated groups on the CB surface, such as 1225 cm^−1^ and 1074 cm^−1^ (C-O-C in highly stable cyclic ethers [43,46]) or 1398 cm^−1^ (C=O in carbonyl and carboxylic compounds [44,45]). The addition of graphite in CB composites did not lead to rise of new bands, the most important effect appearing to be a partial splitting of the band between 1700 cm^−1^ and 1420 cm^−1^, an effect that is more clearly observed in samples with a higher CB content.

### 4.2. Electrical Properties

#### 4.2.1. DC Conductivity

**Figure 7:**
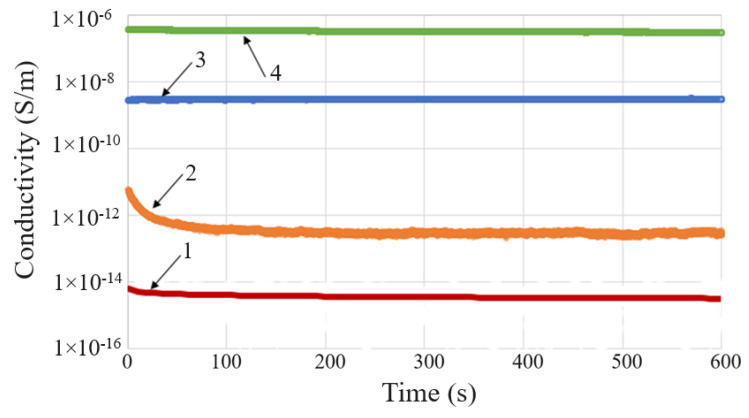
σ_DC_ vs. time, measured at 1 V for LLD neat samples (1) and LLD 80 (2), and at 100 V for LLD 44 (3) and LLD 82 (4).

**Figure 8:**
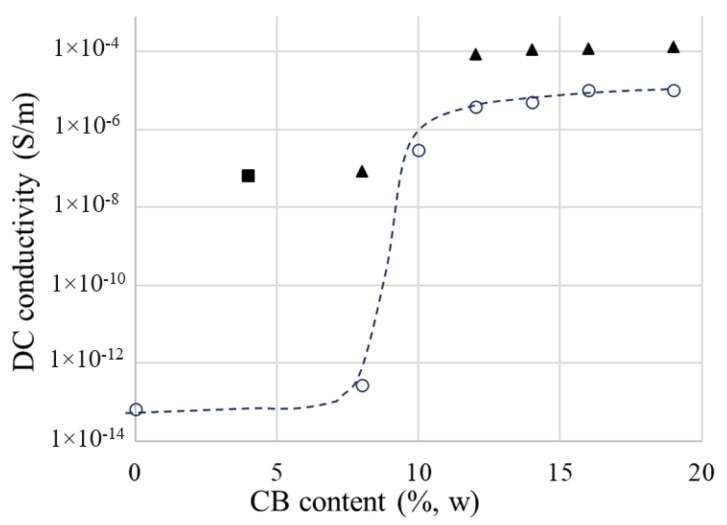
DC conductivity vs. carbon black and graphite content of the studied composites: (○) CB-containing samples; (▲) CB + 2% graphite; (■) CB + 4% graphite.

It was observed that the DC conductivity (σ_DC_) values depend on both the values and durations of the voltage application (Figure 7). In addition, the σ_DC_ values depend on filler concentration (see Figure 8 for CB) and temperature (see Figure S2). The influence of another important factor, namely the molding pressure, will be the subject of a future paper. This paper refers only to the results at 6 bars, a value experimentally found as the minimum required to impart adequate conduction and PTC properties.

In Figure 8, which illustrates the influence of the CB content (ϕ_CB_) on the DC conductivity of CB/LLD composites, three regions can be observed, namely: (i) for low concentrations between 0 and 8%, when σ_dc_ increases slowly with ϕ_CB_, (ii) between 8 and 10%, σ_dc_ increases sharply with a slight change in ϕ_CB_, and (iii) the relatively slow growth domain of σ_dc_ after the jump. The critical concentration, i.e., the minimum concentration at which the composite becomes a conductor (ϕ_c_), corresponds to the jump (ii) and is, in our case, approximately 8%.

The partial substitution of CB with graphite, or the addition of Gr to a certain composition, resulted in the increased conductivity of the composites. Therefore, considering samples LLD 44 and LLD 80, both having a same total content of carbon materials, i.e., 8% (w), the conductivity of the sample with Gr was 68% higher than that of the sample with CB, when is measured at 1 min from the application of the voltage (1 V), and 35% higher when is measured at 10 min. The higher σ_DC_ values observed with the samples containing graphite are related to the considerably higher electrical conductivity of graphite as compared to CB [33], as well as to the morphological characteristics of Gr (high aspect ratio values), which make the transport of charge carriers through the sample easier.

A slight influence of temperature on conductivity was also observed in the range of 30–50 °C. In essence, both the blank sample and the composites with low filler content (i.e., with low values of conductivity) showed a slight decrease in conductivity with time and temperature, which could be due to the extinction of charge carriers generated within the materials during their processing. The materials with higher carbon filler content showed a slight increase in conductivity over time and with temperature (see Figure S2, Table S3), possibly due to a thermally and/or electrically activated local alignment of conductive particles, which could result in a slight increase in the number of conductive paths. Such effects could prevail at some point over the matrix dilatation effect, leading to fluctuations in the monotonous increase in resistivity with temperature, especially at lower temperatures (close to r.t.) and at moderate filler concentrations. The intensity of matrix dilatation effects tends to increase with both the temperature and the conductivity of the sample, as indicated by the R vs. T curves, which become smoother as the resistivity of the material decreases. The fluctuation trend in resistance values with increasing temperature is particularly visible with the LLD 100 and LLD 120 samples.

#### 4.2.2. AC Conductivity

Similarly, to DC conductivity, AC conductivity increases with the content of conductive filler ϕ. This increase is mainly attributed to the growth in the number and dimensions of conductive paths.

**Figure 9:**
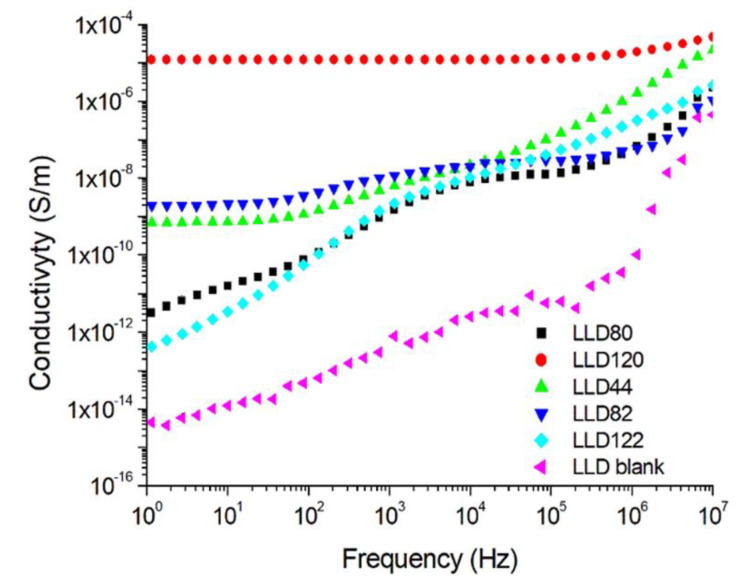
Variation of AC conductivity with the frequency of the measuring voltage for different composites with an LLDPE matrix (U = 1 V).

As depicted in Figure 9, for all samples, the values of σ_ca_ rise with the increase in the frequency of the measuring voltage. A similar behavior was reported for other polymer (nano)composites [34,47]. For example, an increase in frequency from 50 Hz to 1 kHz leads to a 29.5-fold increase in σ_ca_ for the LLD 80 sample and a 7.5-fold increase for LLD 122. In the case of the LLD 120 samples, which had the highest CB content among the analyzed samples, σ_ca_ practically did not vary with the frequency (Figure 9, curve 5). The increase in AC conductivity with frequency can be assigned to charge carrier hopping within the symmetric hopping model in solids with microscopic disorder [34].

**Figure 10:**
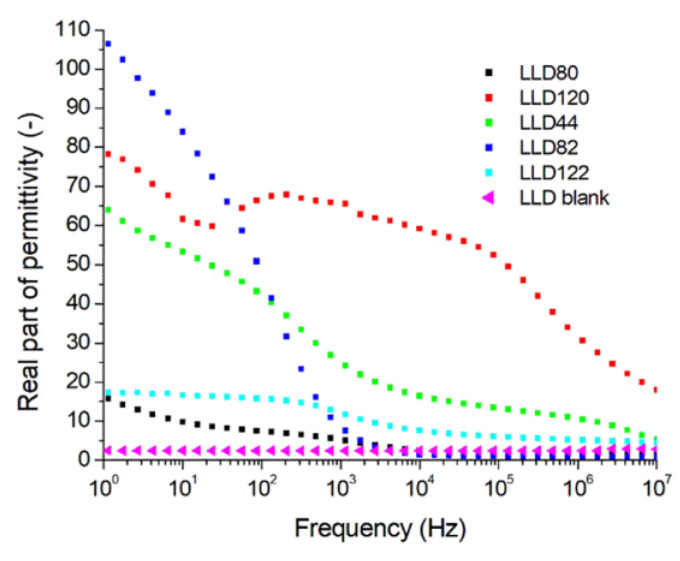
Variation with frequency of the real part of the relative complex permittivity for different composites with an LLDPE matrix (U = 1 V).

The measurement of complex relative permittivity (*ε*_r_′) as a function of frequency revealed (Figure 10) that, in the presence of a conductive filler, the values of *ε*_r_′ are higher compared to the unfilled sample (neat), a result consistent with previous reports for other polymers [28]. More significant differences between the filled and the neat samples were observed in the 1 Hz–1 kHz range, while at frequencies close to 10 MHz, these differences become less pronounced. This increase in *ε*_r_′ is primarily attributed to the new interfaces created by the filler particles, leading to the emergence of inhomogeneity polarization. This phenomenon, in turn, intensifies orientation, electronic and ionic polarizations in the polymeric matrix, resulting in an increase in *ε*_r_′ values. On the other hand, a high filler content also increases the number of clusters and reduces the number of individual carbon black particles dispersed in the polymeric matrix. Consequently, for *ϕ* values greater than 10%, the matrix/filler interface areas decrease, leading to a reduction in *ε*_r_′ values [1,34]. This phenomenon may explain the position of the LLD122 curve on the diagram in Figure 10.

**Figure 11:**
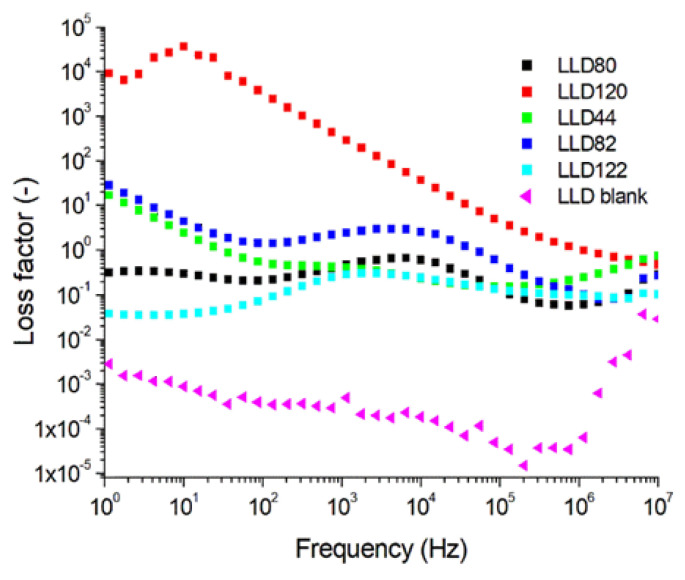
The variation with frequency of the loss factor for LLD neat samples (U = 1 V).

The values of the loss tangent (tan δ) depended on frequency, as shown in Figure 11, exhibiting a trend to increase with the rise in the content of conductive particles, driven by losses due to inhomogeneity polarization.

### 4.3. Resistivity (Resistance) vs. Temperature (PTC Behavior)

The R vs. T curves of samples with carbon contents higher than the critical concentration of 8% (as resulted from the DC conductivity measurements, see above), are similar regardless of the carbon material concentration. These curves present the three typical regions described in the literature (see for example [16]) for PTC composites, namely: (i) a slow increase in resistivity at the beginning of heating from room temperature; (ii) sudden increase of resistivity at elevated temperatures near the polymer melting temperature and reaching a maximum in the proximity of the crystallinity melting temperature (T_m_) of the polymer; and (iii) a gradual decrease in resistivity after reaching the resistivity maximum (NTC effect in the molten state). The interpretation of the phenomena involved in processes (i) and (ii) is usually based on the concept of the thermal expansion of the polymer matrix, which results in increased space between the conductive particles and a gradual disruption of the conductive paths (depercolation). Near a crystalline transition point, the thermally induced volume expansion abruptly increases, causing a resistivity jump, an effect that enables various practical applications, as mentioned above. The decrease in resistivity after reaching the resistivity maximum (NTC effect) is attributed to the formation of conductive aggregates that enable charge carriers to move through the molten polymer [19].

**Figure 12:**
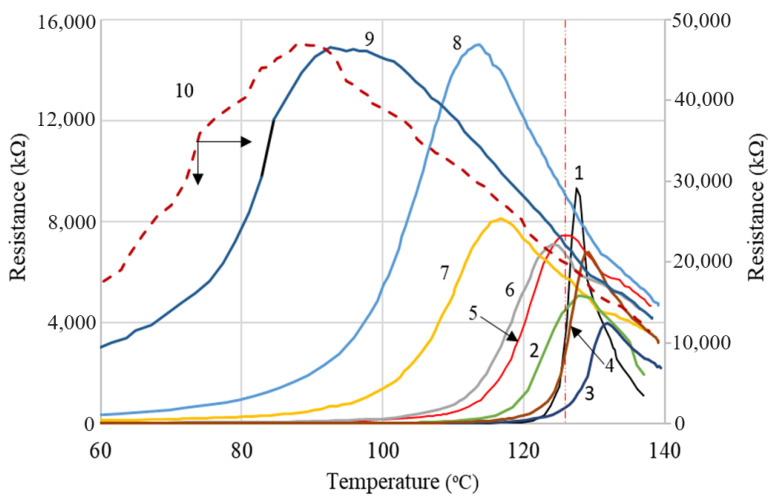
R vs. T curves from (CB, Gr)/LLDPE composites at first heating cycle: 1—LLD 192; 2—LLD 190; 3—LLD 162; 4—LLD 160; 5—LLD 142; 6—LLD 140; 7—LLD 122; 8—LLD 82; 9—LLD 120; 10—LLD 100. The vertical red line correspond to the average T_m(DSC)_ of the composites (125.5 °C).

Although the shape of the R vs. T curves of (CB, Gr)/LLDPE composites follows the general pattern described above, a strong influence of the conductive filler content on the parameters of these curves has been observed (Figure 12). Thus, the onset temperatures (T_onset_) and those of reaching the resistivity maximum (T_max_) decreased as the conductive filler content decreased. Also, the R_max_ values increased as the filler content decreased (see Table S4).

This strong dependence of the R vs. T curves on filler concentration is different from that observed with similar composites with an HDPE-based matrix and seems to be determined by the low crystallinity content of LLDPE. Hence, only for the samples with high conductive filler content, the T_max_ values are close to those corresponding to the melting of the crystalline phase of LLDPE (T_m_) as measured by DSC. Unlike T_max(R)_, T_m(DSC)_ varies very little with the conductive filler concentration, as already mentioned (see Figure 3 and Figure 4 and Table S1). This effect of resistivity on filler concentration was not observed or was insignificant in the case of (CB, Gr)/(LLDPE + HDPE) composites [31]. However, Zhang P et al. [18] reported a dependence of the *ρ* vs. T curves on the filler concentration for the Gr/(LLDPE + HDPE) system, for filler concentrations of 35–50% (mass), but the *ρ* vs. T curves of these materials approach the ideal curve from the literature only for high graphite contents ≥ 40% (for HDPE: LLDPE 1:1) or for LLDPE: HDPE ratios ≥ 60:40. Furthermore, the variation of T_max(R)_ with the filler concentration seems to be rather small. The large difference observed between the values of T_max(DSC)_ and T_max(R)_ was explained by the complex contribution of the total crystallinity of the polymer matrix and the specific volume dilatation [14]. However, the PTC intensity seems to be rather low, with the highest values of the log_10_(*ρ*_max_/*ρ*_0_) ratio being slightly below 3, despite the large graphite concentrations used. The study presented by Zhang R. et al. [12] concerning the influence of carbon fiber (CF) concentration on the *ρ*_DC_ vs. T curves of CF/(UHMWPE+LDPE) composites revealed a behavior similar to that highlighted by us: the onset temperature of the PTC effect decreased considerably (50–123 °C) with the filler content (2.5–10% vol), showing an optimal value (maximum PTC) for 5% CF (vol). The peak temperature of the melting endotherm practically did not change with the CF concentration, but the SEM morphological information suggests rather a homogeneous structure of the matrix and a homogeneous dispersion of CF between the two polymers.

The optimal values of the concentration of conductive fillers, for which the highest intensities of the PTC effect were obtained (Table S4), correspond to samples LLD 140–LLD 190, i.e., those for which the T_max_ values approach the value of the crystallinity melting temperature determined by DSC (T_m(DSC)_). However, it should be noted that all conductive samples showed significant jumps in resistivity with increasing temperature. The samples with high r.t resistivity (LLD 100, LLD 122 and LLD 82) exhibit broad peaks of resistivity, which occur at considerably lower temperatures than the melting temperature of the polymer. Combining the experimental observations and the data from the literature, it can be concluded that the behavior of the studied composites in R vs. T measurements could be described more properly by the Ohe and Natio model [34,35], based on tunneling through the interparticle gaps, rather than the model based on sudden expansion at the melting temperature. Thus, for samples with a lower filler content, depercolation occurs as a result of tunneling interruption due to the thermally induced modification of the space gap distribution between the conductive particles (a contribution of molecular movements or the melting of pseudo-crystalline domains is to be also considered). On the other hand, at high concentrations of the conductive filler, a more significant volumetric expansion is necessary (like what is produced near the crystallinity melting temperature), to sufficiently increase the distances between conductive particles; hence, a mechanism based on thermal expansion predominates in such a case.

If we compare the values of T_m(DSC)_ and T_max(R)_ for a same sample, it can be observed that excluding the samples with a high carbon contents (LLD 192, LLD 190, LLD 162 and LLD 160), the reaching of the R_max_ value and the subsequent decrease upon heating occur in the “solid” phase, at lower temperatures than T_m(DSC)_. This fact suggests that the aggregates of conductive particles, considered responsible for the decrease in resistivity after R_max_, are formed even before complete polymer melting and that the mobility of these aggregates within the polymer matrix is sufficiently high to ensure electrical conduction. Therefore, the enhancement of electrical conduction at T_max(DSC)_ > T > T_max(R)_ could be provided by a thermally activated mechanism of jumping/tunneling between neighboring conductive (fibrous) aggregates, which could result from the coagulation (re-arrangement) of the neighboring conductive particles. Note that the viscosity of the medium at elevated temperatures (below the melting point) is high enough to enable only the limited movement of the conductive particles around their equilibrium positions in the conductive paths. This could also explain the observed broadening of the resistivity peak (which equals to a decrease in the intensity of the NTC effect) with decreasing conductive filler concentrations.

If we compare the values of T_m(DSC)_ and T_max(R)_ for the same sample, it can be observed that excluding the samples with high carbon contents (LLD 192, LLD 190, LLD 162 and LLD 160), the R_max_ value is reached well before the matrix melts and the subsequent decrease occurs in the “solid” phase until a temperature equal to T_m(DSC)_ is attained. This fact suggests that aggregates of the conductive particles, considered to be responsible for the decrease in resistivity after R_max_, are formed well before complete polymer melting, and that the mobility of these aggregates within the polymer matrix is sufficiently high to ensure electrical conduction. Therefore, the decrease in electrical resistivity at T_m(DSC)_ > T > T_max(R)_ could be provided by a thermally activated mechanism of jumping/tunneling between neighboring conductive (fibrous) aggregates, which may result from the coagulation (re-arrangement) of the neighboring conductive particles. Note that the viscosity of the medium at elevated temperatures (below the melting point) is high enough to enable only limited movement of the conductive particles around their equilibrium positions in the conductive paths. This fact could also explain the observed broadening of the resistivity peak (which equals to a decrease in the intensity of the NTC effect) with decreasing conductive filler concentrations.

**Figure 13:**
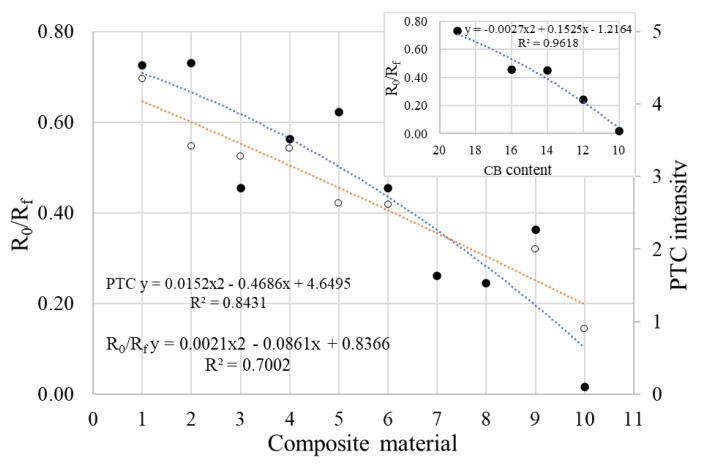
*R_0_/R_f_* (•) and *PTC* intensity (○) of different composites (see numbers on the abscise): 1—LLD 192; 2—LLD 190; 3—LLD 162; 4—LLD 160; 5—LLD 142; 6—LLD 140; 7—LLD 122; 8—LLD 120; 9—LLD 82; 10—LLD 100. In the inset, decreasing R_0_/R_f_ values for CB/LLDPE composites as CB content decreases.

**Figure 14:**
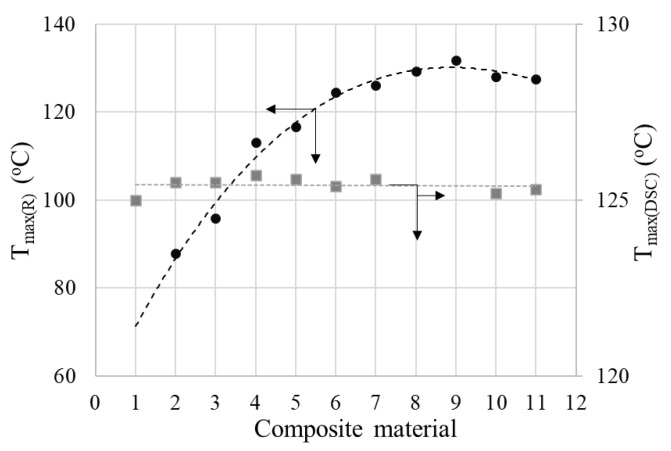
*T_max(R)_* and *T_max(DSC)_* vs. carbon content of composites (see numbers on the abscise): 1—LLD 0; 2—LLD 100; 3—LLD 82; 4—LLD 120; 5—LLD 122; 6—LLD 140; 7—LLD 142; 8—LLD 160; 9—LLD 162; 10—LLD 190; 11—LLD 192.

Additionally, it is observed from the comparison of R vs. T and DSC data (Figure 12, Figure 13 and Figure 14) that even for samples with a high carbon content, there is no perfect correspondence between the T_max(R)_ and T_max(DSC)_ values. For some of the samples mentioned above as having a high carbon content, the T_max(R)_ values are slightly higher than the T_m(DSC)_, although the heating rates in the R vs. T measurements were lower. This behavior suggests that the same distribution of particles that exists in the solid state persists in the highly viscous fluid material that resulted from the crystallinity melting. On the other hand, the attainment of R_max_ before the crystallinity melting for the samples with a lower carbon content suggests that the smaller volume expansion of the amorphous phase at T < T_m(DSC)_ would be large enough to increase the interparticle distances interrupting so the conductive paths in those samples.

The decrease in resistivity after T_max(R)_ could be explained by the formation of aggregates in the amorphous zone of the not yet melted polymer, through a continuous process apparently unaffected by the melting of the polymer (the shape of the R vs. T curves did not change dramatically in the proximity of T_m(DSC)_). In understanding the behavior of the composites below T_m(DSC)_, we should take into account that the conductive material is distributed in the amorphous region of the polymer (see, e.g., Zhang P et al. [19]) and that the composite preparation method provides a certain degree of inhomogeneity in the conductive particles distribution. Hence it seems that it can be expected that the changes in volume and viscosity in either the amorphous or pseudo-crystalline phases and/or the activation of some thermally induced molecular movements [19] influence the electrical conductivity of the material more than the melting crystallinity, which is present in a relatively small proportion, according to the DSC data (see the Supplementary Materials). In particular, in the case of LLDPE, which exhibits a complicated behavior during melting and solidification [48], such processes can influence the distribution of conductive particles and, consequently, the resistivity of the material during heating.

The analysis of the data in Tables S1 and S4 and in Figure 13 and Figure 14 suggests a relatively complex influence of the carbon content on the R-T behavior: according to the Tmax(R) values, the composites can be divided into two groups, namely (i) samples with a high total carbon content (≥14% *w*/*w*) which present T_max(R)_ values close to T_m(DSC)_ and (ii) samples with a low total carbon content (≤12% *w*/*w*), for which the difference in T_mDSC_ − T_max(R)_ increases as the carbon content decreases. The intensities of the PTC effect have high values in the first group and decrease as the carbon content of the sample decreases. The presence of graphite generally improved the parameters of the R-T curves parameters, but the most noticeable effect was observed at low total carbon concentrations, as seen in the case of sample LLD 82, which exhibits comparable parameters to sample LLD 122, despite having a lower carbon content. Additionally, the differences in behavior induced by the presence of graphite are clear if we compare samples LLD 122 vs. LLD 120, or LLD 82 vs. LLD 100. In addition, it shall be remarked that these results are in good agreement with the AC and DC conductivity data presented above.

**Figure 15:**
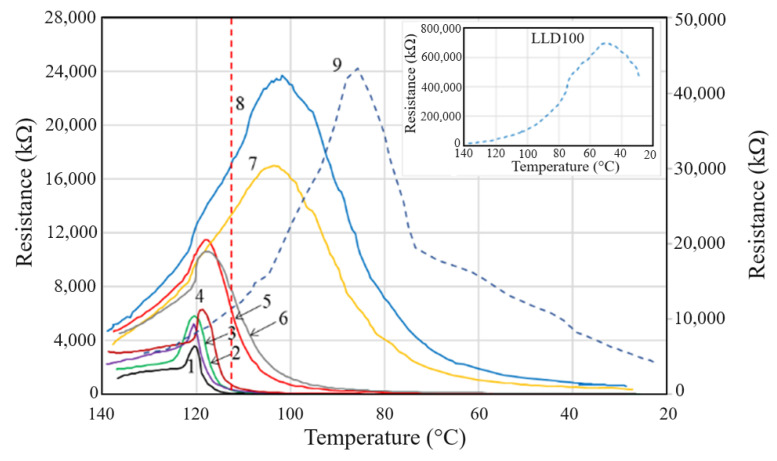
R vs. T curves from (CB, Gr)/LLDPE composites at first cooling cycle: 1—LLD 192; 2—LLD 190; 3—LLD 162; 4—LLD 160; 5—LLD 142; 6—LLD 140; 7—LLD 122; 8—LLD 82; 9—LLD 120. In inset, LLD 100 sample (note the max. of y scale is 800,000 kΩ). The vertical red line corresponds to average T_c_ of the composites (112.8 °C).

In the case of cooling curves (Figure 15), similar trends were observed, but the values of R’_max_ are higher than the corresponding R_max_ values from the heating curves (excluding sample LLD 192), suggesting a slower reformation of conductive paths upon solidification.

Overall, all samples showed higher resistivity values at room temperature after a heating–cooling cycle, indicating that some of the conductive pathways do not reform. Considering a proportional relationship between the number of conductive pathways and conductivity (inverse of resistivity), Figure 13 shows that the fraction of channels that do not reform (described by R_0_/R_f_ ratio) is smaller in samples with a high carbonaceous conductive filler content (>14 *w*/*w*) and increases with the decreasing carbon content of the samples. The scattering of the R_0_/R_f_ values seems to be influenced by the graphite-containing samples, where the variation trend is weaker than for the samples containing CB only (see Figure 13 inset).

The conductive samples with lower filler concentrations (at the end of the conductive range, i.e., LLD 82–LLD 192) exhibit a different behavior during cooling as compared to other samples in the mentioned range. Thus, the LLD 120 and LLD 100 samples, with a relatively low concentration of carbon black and no graphite, return to high resistance values after cooling, in the order of MΩ and hundreds of MΩ, respectively, categorizing them as insulators; hence, the R_0_/R_f_ values are low. The behavior of the LLD 192 sample, which contains a high concentration of fillers, is remarkable too: the cooling curves (see Figure 15 and Table S5) show a slow increase in melt resistivity between 133 and 123 °C, after which the resistivity suddenly increases until ~120 °C (T′_max(R)_). This value is far from T_c(DSC)_ (see Figure 15 and Table S2). However, the maximum value (R’_max_) is considerably lower than the corresponding value from the heating curves, representing a different behavior not only compared to the rest of the studied composites, but also in comparison to the (Gr, CB)/HDPE and (Gr, CB)/(HDPE + LLDPE) composites reported earlier [31]. This behavior indicates that the material conserves relatively high conductivity at the crystallization temperature, i.e., a significant number of the conductive pathways remain functional at T′_max(R)_.

In general, it is observed (Figure 15 and Tables S2 and S5) that all samples with high carbon content (LLD 140–LLD 192) have T′_max(R)_ values higher than T_c(DSC)_, with little dependence on filler concentration, while the samples with lower conductive filler content present lower T′_max(R)_ values (and are strongly dependent on the filler concentration) than T_c(DSC)_.

### 4.4. Electrothermal Behavior

**Figure 16:**
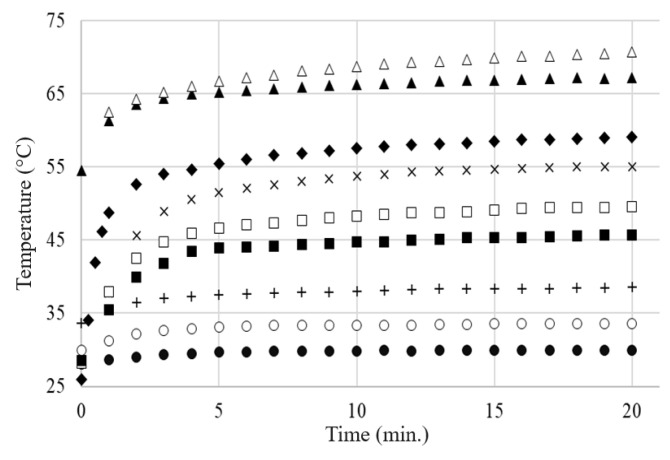
The *T* vs. *t* curves for different applied voltages (AC) on the same composite sample LLD 192 (s10): (●) 3 V; (○) 5 V; (+) 8 V; (■) 10 V; (☐) 12 V; (×) 15 V; (▲) 20 V; (∆) 25 V; (♦) 35 V Levelling of temperature increase at 20 and 25 V can be clearly observed.

**Figure 17:**
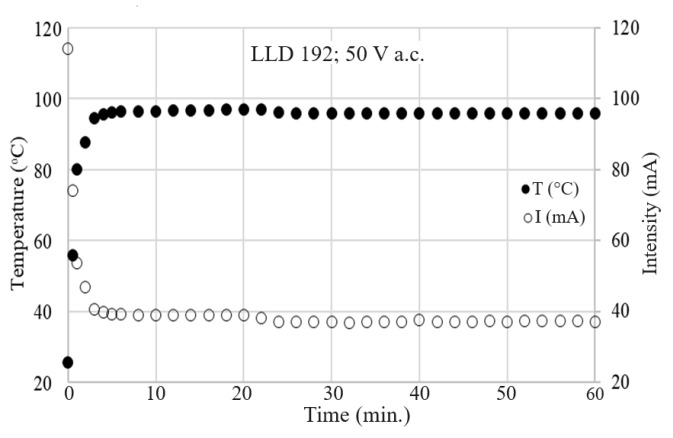
The T vs. t and I vs. t curves for LLD 192 sample (U = 50 V).

For all samples with a higher carbon content than the critical concentration, regardless of the carbon content and applied voltage, the temperature–time variation (T vs. t) curves on the sample surface exhibit a typical self-limiting behavior of temperature and current, enabling the use of these materials in self-regulating thermal applications (Figure 16 and Figure 17).

**Figure 18:**
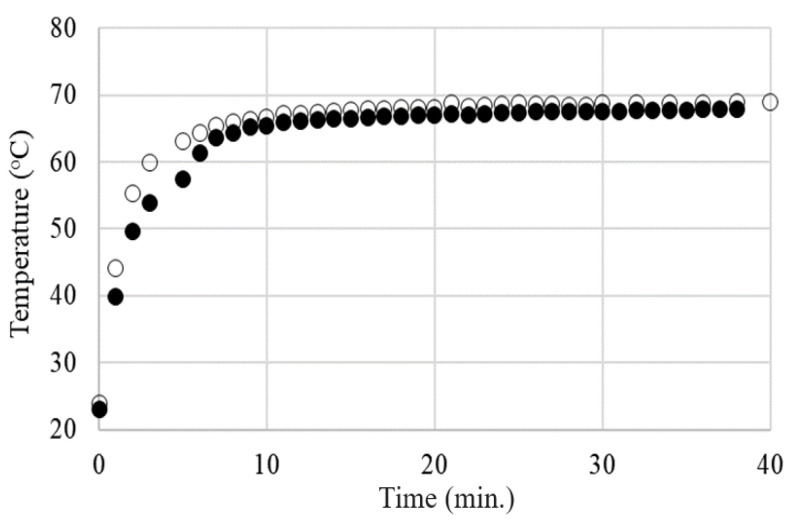
The T vs. t curves for LLD 192 at 20 V: (○) DC; (●) AC.

No significant differences were observed between the T vs. t curves resulting from the DC and AC measurements for the same applied voltages (effective voltage in AC), as can be observed in Figure 18 for the LLD 192 sample at applied voltages of 20 V.

**Figure 19:**
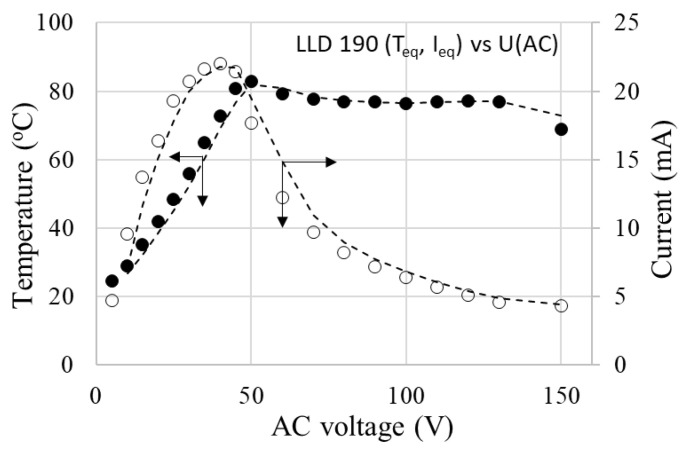
T_eq_ vs. applied voltage for LLD 190 sample.

The voltage-limiting effect is illustrated in Figure 19 for the LLD 190 sample. In the low voltage range, up to 50 V in this case, the temperature increased with increasing voltage, reaching a maximum. Subsequently, the equilibrium temperature values decreased with increasing voltage. This trend was accentuated as the applied voltage increased. Such a behavior is of particular practical importance because it describes overvoltage protection.

It is noteworthy that the Teq value decreased with the decreasing of the conductive filler concentration (or, for achieving the same temperature, the necessary voltage was higher as the concentration of the conductive filler was lower).

Also, for any of the sample combinations (filler concentration)—the applied voltage, the maximum value of T_eq_ was far enough from T_m(DSC)_, meaning that the temperature limitation occurs at considerably lower T_eq_ values. This fact is also of practical importance for the operational safety of the material as a heater, preventing its destruction by melting during use. Additionally, because the Teq values differ significantly from the T_m(DSC)_ values, our previous observations concerning the limited involvement of the crystalline–amorphous transition in the PTC effect mechanism in the case of this type of polymer matrix are confirmed (see R vs. T curves).

The dependency of the thermal–voltage effect described above, observed in all of the studied samples, differs significantly from the behavior of an ohmic resistor and is determined by the self-limiting characteristics of the material. If the self-limiting effect did not occur, then the current intensity would increase proportionally with the applied voltage, following Ohm’s law (U = RI, with R practically constant). However, in our case, it is clearly observed (see Figure 19) that the equilibrium current intensity through the sample decreases with the applied voltage. This effect directly affects the temperature on the surface of the sample, which remains unchanged over time for a given voltage (Figure 17) and changes very little over a wide range of voltages (Figure 19). At the same time, the decrease in I_eq_ with applied voltage suggests that as the voltage tends toward infinity, the material would tend to become an insulator (Figure 19) due to the self-limitation of the current passing through the sample.

It was also observed, especially for samples exposed to high AC voltages (>150 °C), that a slight increase in r.t. resistivity (measured as resistance) occurred after conducting long term tests. Although, after exposure to moderate voltages (25–50 V), the resistivity tends to decrease slightly, suggesting the reversibility of the process, and the possible aging of the polymeric material (or the matrix–filler assembly) at elevated temperatures and high voltages cannot be excluded. Hence, the extension of the work in the direction of degradation diagnosis is considered.

## 5. Conclusions

This study reaffirms the significant influence of filler content (ϕ) on the electrical properties of polyethylene/carbon black/graphite composites. A notable increase in DC conductivity was observed between 8–10% filler content due to percolation. Higher ϕ values also increased AC conductivity, permittivity and the loss factor, leading to enhanced dielectric losses and subsequent heating in electric fields.

The use of an LLDPE matrix with its low crystallinity offers greater flexibility compared to HDPE. A strong dependence of the PTC effect on carbon content was demonstrated, particularly on the temperature at which maximum resistivity occurs (T_max(R)_). This effect is underexplored in the literature and highlights a unique aspect of this study.

The results revealed how the composition of a composite significantly impacts self-limiting properties and the PTC effect. Above the critical percolation concentration, there exists an optimal range where the PTC effect’s intensity and stability hold practical value for (CB-Gr)/LLDPE composites. Unlike highly crystalline matrices (HDPE or HDPE/LLDPE), T_max(R)_ and the DSC-determined melting temperature (T_m(DSC)_) differ significantly. This indicates that factors beyond crystallinity melting influence the PTC mechanism. Further studies are needed to explore the specific interaction between the conductive filler and the LLDPE matrix.

The distinct behavior of LLDPE-matrix composites allows for the tailored control of self-limiting and interrupting properties, potentially leading to innovative low-temperature applications.

## Figures and Tables

**Table 1:** List of abbreviations and symbols.

Abbreviation	Full Name/Description
AC	Alternating Current
CB	Carbon Black
(CB, Gr)/LLD	Composites with LLDPE matrix and CB and Gr
CF	Carbon Fibers
CPC	Conductive Polymer Composite
DC	Direct Current
DSC	Differential Scanning Calorimetry
ΔH	Transition enthalpy (from DSC)
ε′, ε″	complex relative permitivities (AC)
FTIR	Fourier Transform InfraRed spectroscopy
ϕ	Fraction (either mass or volume) of conductive filler within the composite
ϕ_c_	Critical concentration of the filler
Gr	Graphite
HDPE	High Density Polyethylene
LDPE	Low Density Polyethylene
LLDPE	Linear Low-Density Polyethylene
NTC	Negative Temperature Coefficient
PTC	Positive Temperature Coefficient
*R*	Electrical resistance
*ρ*	Electrical resistivity
*ρ_DC_*	Direct current resistivity
*ρ_V_*	Volume resistivity
RMS voltage	Root Mean Square voltage (effective voltage = 0.707 of peak voltage, in AC measurements)
*σ′*, *σ″*	complex conductivities (AC)
*σ_dc_*	Direct current conductivity
*SEM*	Scanning Electron Microscopy
*T_c_*, *T_c(DSC)_* (see the footnote for T_m)_	peak temperature of crystallization endotherm (in DSC)
*Teq*	Equilibrium temperature (T_eq_ denotes the practically constant value of the surface temperature reached after few minutes of sample exposure to electric field, in T vs. t, U measurements)
*T_m_; T_m(DSC)_* (The notation T_m(DSC)_ is used for better distinguish between the maximum of temperatures in either DSC and R vs. T measurements)	Peak temperature of crystallinity melting in DSC
*T_offset_*	Offset temperature in DSC or *R* vs. *T-heating* measurements
*T′offset*	Offset temperature in *R* vs. *T-cooling* measurements
*T_onset_*	Onset temperature in DSC or *R* vs. *T-heating* measurements
UHMWPE	Ultra-High Molecular Weight Polyethylene

**Table 2:** Code names and composition of the studied samples.

Sample Code	Polymeric Matrix	Filler		Total C (%, w)
	LLDPE (%, w)	Carbon Black (%, w)	Graphite (%, w)	
LLD 0	100 (neat)	0	0	0
LLD 44	92	4	4	8
LLD 80	92	8	0	8
LLD 82	90	8	2	10
LLD 100	90	10	0	10
LLD 120	88	12	0	12
LLD 122	86	12	2	14
LLD 140	86	14	0	14
LLD 142	84	14	2	16
LLD 160	84	16	0	16
LLD 162	82	16	2	18
LLD 190	81	19	0	19
LLD 192	79	19	2	21

## Data Availability

Data are contained within the article and Supplementary Materials.

## Supplementary Materials

The following supporting information can be downloaded at: https://www.mdpi.com/article/10.3390/ma17051224/s1, Figure S1: Theoretical curve of DC conductivity vs. conductive filler concentration; Figure S2: Electrical conductivity (σ_DC_) vs. time for LLD 122 sample, at different temperatures: (1) 30 °C; (2) 40 °C; (3) 50 °C. The measurement voltage, U0 = 1V; Table S1: Kinetic parameters of melting process studied by DSC (ramp experiment, heating rate, 10 °C/min., N2 flow, 50 ml/min); Table S2: Kinetic parameters of crystallization process studied by DSC (ramp experiment, cooling rate, 10 °C/min., N2 flow, 50 ml/min); Table S3: *σ_DC_* values measured at different temperatures (*T*) after 1 min. (σ*_dc_*_1_) and 10 min. (σ*_dc_*_10_) from voltage (*U_0_*) application; Table S4: Kinetic parameters of the *R vs. T* heating curves of the studied composites; Table S5: Kinetic parameters of the R vs. T cooling curves of the studied composites.
