# Supplementary material for: Electrical and Electro-Thermal Characteristics of (Carbon Black-Graphite)/LLDPE Composites with PTC Effect
Source: Materials (Basel). 2024 Mar 6;17(5):1224. doi: 10.3390/ma17051224 (PMC10935172; doi:10.3390/ma17051224)
Supplement: Supplementary file 1 [file materials-17-01224-s001.zip › materials-2871256-supplementary.pdf]

SUPPLEMENTARY MATERIAL

Electrical and Electro-Thermal Characteristics of (Carbon Black-Graphite)/LLDPE Composites with PTC Effect

Eduard-Marius Lungulescu [1,*], Cristina Stancu [2,*], Radu Setnescu [1,3], Petru V. Notingher [2] and Teodor-Adrian Badea [4]

1 National Institute for Research and Development in Electrical Engineering ICPE-CA, 313 Splaiul Unirii, 030138 Bucharest, Romania
2 Faculty of Electrical Engineering, University POLITEHNICA of Bucharest, 313 Splaiul Independentei, 060042 Bucharest, Romania
3 Department of Advanced Technologies, Faculty of Sciences and Arts, Valahia University of Târgoviște, 13 Aleea Sinaia, 130004 Targoviste, Romania
4 Romanian Research and Development Institute for Gas Turbines COMOTI, 220D Iuliu Maniu Av., 061126 Bucharest, Romania
* Correspondence: marius.lungulescu@icpe-ca.ro (E.-M.L.); stcris2003@yahoo.co.uk (C.S.)

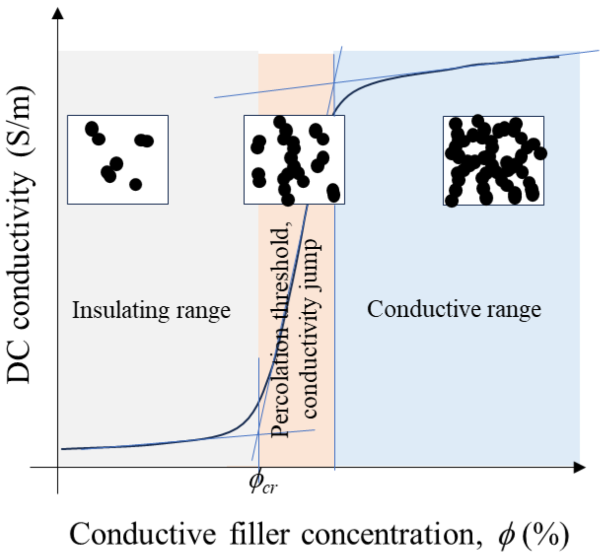

Figure S1. Theoretical curve of DC conductivity vs. conductive filler concentration

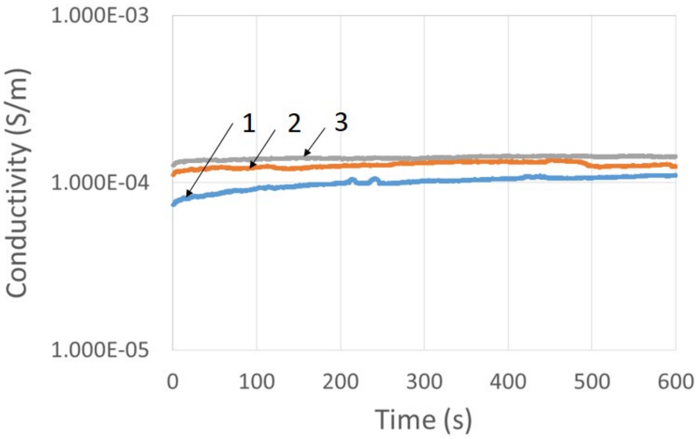

Figure S2. Electrical conductivity (σ_{DC}) vs time for LLD 122 sample, at different temperatures: (1) 30 °C; (2) 40 °C; (3) 50 °C. The measurement voltage, U_0 = 1V

Table S1. Kinetic parameters of melting process studied by DSC (ramp experiment, heating rate, 10 ^{o}C/min., N_2 flow, 50 ml/min)

Sample	Mass (mg)	DSC run	*p*(%) LLDPE	*ΔH* (J/g)	ΔH_{corr} (J/g)	*Cr*(%)	T_m (^{o}C)	T_{onset} (^{o}C)	T_{offset} (^{o}C)
LLD 0	3.6	1	100	136.3	136.3	48.9	125.8	120.0	128.4
		2	100	117.8	117.8	42.2	125.0	121.2	127.5
LLD 82	4.5	1	90	122.9	136.6	49.0	128.3	119.7	131.7
		2	90	105.1	116.8	41.9	125.7	119.9	128.9
LLD 100	4.5	1	90	120.4	133.8	47.9	126.9	123.9	129.6
		2	90	106.8	118.7	42.5	125.5	121.0	128.2
LLD 120	4.6	1	88	116.6	132.5	42.5	125.8	119.3	128.6
		2	88	102.6	116.6	41.8	125.5	120.3	128.3
LLD 122	4.6	1	86	111.6	129.8	46.5	127.1	120.4	132.5
		2	86	100.1	116.4	41.7	125.6	119.8	128.9
LLD 140	4.6	1	86	116.0	134.9	48.3	127.8	120.1	131.2
		2	86	98.8	114.9	41.2	125.4	120.8	128.1
LLD 142	4.4	1	84	114.8	136.7	49.0	128.1	120.3	131.6
		2	84	98.0	116.7	41.8	125.6	120.3	128.8
LLD 190	4.5	1	81	93.7	115.7	41.5	127.5	120.6	130.8
		2	81	91.7	113.2	40.6	125.2	120.9	127.8
LLD 192	4.2	1	79	108.0	136.7	49.0	127.7	119.8	131.4
		2	79	93.1	117.8	42.2	125.3	120.0	128.5

Table S2. Kinetic parameters of crystallization process studied by DSC (ramp experiment, cooling rate, 10 ^{o}C/min., N_2 flow, 50 ml/min)

Sample	Mass (mg)	DSC run	*p*(%) LLDPE	*ΔH* (J/g)	ΔH_{corr} (J/g)	*Cr*(%)	T_m (^{o}C)	T_{onset} (^{o}C)	T_{offset} (^{o}C)
LLD 0	3.6	1	100	-117.3	-117.3	42.0	111.1	113.4	107.1
		2	100	-118.0	-118.0	42.3	111.2	113.4	107.3
LLD 82	4.5	1	90	-103.6	-115.1	41.3	113.9	116.7	106.5
		2	90	-103.2	-114.7	39.7	113.8	116.8	107.2
LLD 100	4.5	1	90	-104.2	-115.7	41.5	112.6	114.8	107.6
		2	90	-104.1	-115.7	41.5	112.6	114.9	107.8
LLD 120	4.6	1	88	-102.3	-116.3	41.7	113.0	115.3	108.1
		2	88	-102.2	-116.1	41.6	112.9	115.2	107.4
LLD 122	4.6	1	86	-100.2	-116.5	41.8	113.5	116.6	106.6
		2	86	-100.2	-116.5	41.8	113.4	116.7	107.3
LLD 140	4.6	1	86	-98.9	-115.0	41.2	112.0	114.6	107.0
		2	86	-99.3	-115.5	41.4	112.0	114.7	107.2
LLD 142	4.4	1	84	-98.3	-117.0	41.9	112.6	116.6	106.8
		2	84	-98.1	-116.8	41.9	112.5	116.5	106.8
LLD 190	4.5	1	81	-94.2	-116.3	41.7	112.2	114.5	107.0
		2	81	-94.37	-116.5	41.8	112.2	114.6	107.3
LLD 192	4.2	1	79	-92.98	-117.7	42.2	112.9	116.3	107.4
		2	79	-93.47	-118.3	42.4	112.9	116.3	107.2

Table S3. σ_{DC} values measured at different temperatures (T) after 1 min. (σ_{dc1}) and 10 min. (σ_{dc10}) from voltage (U_0) application

**Sample**	U_0 **(V)**	T **(°C)**	σ_1 **(S/m)**	σ_{10} **(S/m)**
LLD blank	100	30	$0.51\cdot10^{-14}$	$0.16\cdot10^{-14}$
		40	$0.54\cdot10^{-14}$	$0.15\cdot10^{-14}$
		50	$0.85\cdot10^{-15}$	$0.32\cdot10^{-15}$
LLD 82	100	30	$0.85\cdot10^{-7}$	$0.75\cdot10^{-7}$
		40	$0.72\cdot10^{-7}$	$0.63\cdot10^{-7}$
		50	$0.55\cdot10^{-7}$	$0.47\cdot10^{-7}$
LLD 44	100	30	$0.63\cdot10^{-7}$	$0.53\cdot10^{-7}$
		40	$0.50\cdot10^{-7}$	$0.47\cdot10^{-7}$
		50	$0.44\cdot10^{-7}$	$0.36\cdot10^{-7}$
LLD 80	1	30	$0.25\cdot10^{-11}$	$0.13\cdot10^{-11}$
		40	$0.31\cdot10-^{11}$	$0.10\cdot10^{-11}$
		50	$0.20\cdot10^{-12}$	$0.17\cdot10^{-12}$
LLD 100	1	30	$0.21\cdot10^{-5}$	$0.21\cdot10^{-5}$
		40	$0.21\cdot10^{-5}$	$0.22\cdot10^{-5}$
		50	$0.56\cdot10^{-5}$	$0.52\cdot10^{-5}$
LLD 120	1	30	$0.88\cdot10^{-5}$	$0.93\cdot10^{-5}$
		40	$0.97\cdot10^{-5}$	$0.94\cdot10^{-5}$
		50	$0.87\cdot10^{-5}$	$0.96\cdot10^{-5}$
LLD 122	1	30	$0.86\cdot10^{-4}$	$0.11\cdot10^{-3}$
		40	$0.12\cdot10^{-3}$	$0.12\cdot10^{-3}$
		50	$0.13\cdot10^{-3}$	$0.14\cdot10^{-3}$

Table S4. Kinetic parameters of the *R vs T* heating curves of the studied composites

**Sample code**	R_0 **(kΩ)**	T_{onset} **(°C)**	**Slope of** R **increase/**R_{max} **(K⁻¹)**	T_{max} **(°C)**	R_{max} **(kΩ)**	**PTC intensity** $\log_{10}(R_{max}/R_0)$	**Slope of** R **decrease/**R_{max} **(K⁻¹)**	T_{offset} **(°C)**	R_{offset} **(kΩ)**	R_{offset}/R_{max}
LLD 192	0.415	125.1	0.35	127.5	9350	4.35	-0.35; -0.08	129.1	5760	0.62
LLD 190	1.936	118.5	0.14	128.1	5060	3.42	-0.07	-	-	-
LLD 162	2.08	127.4	0.27	131.8	3980	3.28	-0.07	135.9	2790	0.70
LLD 160	2.76	124.1	0.22	129.2	6780	3.39	-0.07; -0.03	134.5	4400	0.56
LLD 142	11.70	116.2	0.09	126.0	7460	2.80	-0.05; -0.02	130.8	6030	0.81
LLD 140	16.97	112.0	0.08	124.4	7100	2.62	-0.04; -0.03	128.0	5950	0.68
LLD 122	69.8	102.1	0.08	116.7	8130	2.07	-0.03; -0.01	131.1	4410	0.54
LLD 120	1270	73.4	0.05	95.8	14830	1.07	-0.02; -0.01	128.6	4190	0.30
LLD 82	150.4	95.2	0.06	113.1	15010	2.00	-0.04; -0.02	131.0	6740	0.45

Table S5. Kinetic parameters of the R vs T cooling curves of the studied composites

**Sample code**	T'_{onset} **(°C)**	**Slopes of** R **increase/**R_{max} **(K⁻¹)**	T'_{max} **(°C)**	R'_{max} **(kΩ)**	**Slope of** R **decrease/**R_{max} **(K⁻¹)**	T'_{offset} **(°C)**	R_f **(kΩ)**	R_0/R_f
LLD 192	122.8	-0.01; -0.21	120.5	3600	0.68	118.2	0.603	0.73
LLD 190	123.8	-0.09; -0.20	120.5	5810	0.26	115.75	2.92	0.73
LLD 162	122.6	-0.015; -0.16	120.5	5240	0.28	116.6	4.92	0.46
LLD 160	120.7	-0.009; -0.37	118.7	6300	0.29	114.5	5.37	0.56
LLD 142	122.8	-0.02; -0.07	117.7	11490	0.11	107.8	27	0.43
LLD 140	120.7	-0.02; -0.27	118.0	10620	0.08	103.5	37.3	0.45
LLD 122	123.8	-0.015, -0.030	103.6	17010	0.04	78.8	292	0.24
LLD 120	118.4	-0.07; 0.05	74.2	44100	0.03	44.7	5210	0.30
LLD 100	94.5	~0.0003; -0.02	52.3	69800	0.015	-	468000	0.02
LLD 82	122.9	-0.01; -0.03	101.8	23700	0.04	75.9	585	0.36
